# Towards an Efficient and Exact Algorithm for Dynamic Dedicated Path Protection

**DOI:** 10.3390/e23091116

**Published:** 2021-08-27

**Authors:** Ireneusz Szcześniak, Ireneusz Olszewski, Bożena Woźna-Szcześniak

**Affiliations:** 1 Department of Computer Science, Częstochowa University of Technology, 42-200 Częstochowa, Poland; 2 Institute of Telecommunications, UTP University of Sciences and Technology, 85-796 Bydgoszcz, Poland; irek@utp.edu.pl; 3 Department of Mathematics and Computer Science, Jan Długosz University, 42-200 Częstochowa, Poland; b.wozna@ujd.edu.pl

**Keywords:** dynamic dedicated path protection, generic Dijkstra algorithm, elastic optical network, modulation constraints

## Abstract

We present a novel algorithm for dynamic routing with dedicated path protection which, as the presented simulation results suggest, can be efficient and exact. We present the algorithm in the setting of optical networks, but it should be applicable to other networks, where services have to be protected, and where the network resources are finite and discrete, e.g., wireless radio or networks capable of advance resource reservation. To the best of our knowledge, we are the first to propose an algorithm for this long-standing fundamental problem, which can be efficient and exact, as suggested by simulation results. The algorithm can be efficient because it can solve large problems, and it can be exact because its results are optimal, as demonstrated and corroborated by simulations. We offer a worst-case analysis to argue that the search space is polynomially upper bounded. Network operations, management, and control require efficient and exact algorithms, especially now, when greater emphasis is placed on network performance, reliability, softwarization, agility, and return on investment. The proposed algorithm uses our generic Dijkstra algorithm on a search graph generated “on-the-fly” based on the input graph. We corroborated the optimality of the results of the proposed algorithm with brute-force enumeration for networks up to 15 nodes large. We present the extensive simulation results of dedicated-path protection with signal modulation constraints for elastic optical networks of 25, 50, and 100 nodes, and with 160, 320, and 640 spectrum units. We also compare the bandwidth blocking probability with the commonly-used edge-exclusion algorithm. We had 48,600 simulation runs with about 41 million searches.

## 1. Introduction

Optical networks, which are the backbone of communication networks, need to provide protection for the carried traffic to prevent large-scale disruptions due to fiber cuts, human errors, hardware failures, power outages, natural disasters or attacks [1,2,3]. From among the various ways of protecting traffic in optical networks, dedicated path protection (DPP) is the simplest, and the most effective, albeit the most expensive. In DPP, there are two paths established for a single demand: the working one, and the protecting one. When the working path fails, the protecting path delivers the traffic. DPP has been commonly used and studied for decades.

In a wavelength-division multiplexed (WDM) network, if a client signal does not fully utilize the fixed spectrum of the assigned wavelength, the spectrum of the precious erbium window is wasted, a problem addressed by elastic optical networks (EONs) which divide the spectrum into fine *frequency slot units* (of, e.g., 12.5 GHz width), or just *units*, and then allocating *contiguous units* to form a *slot* tailored to a specific demand [4].

Routing in WDM networks with the spectrum continuity constraint is called routing and wavelength assignment (RWA). Routing in EONs with the spectrum contiguity constraint added is called routing and spectrum assignment (RSA), and with the signal modulation constraint added is called routing, modulation, and spectrum assignment (RMSA). These routing problems can be dynamic or static. In dynamic (aka online) routing, a single demand is routed in a loaded network, as opposed to *static* (aka offline) routing, where many demands are routed in an unloaded network.

When finding an exact solution for a dynamic routing problem in optical networks, some path cost is minimized, and the spectrum and modulation constraints are met. The path cost can be defined in various ways, e.g., the path length, the number of edges, some signal quality measure, monetary cost, or a measure related to availability. The path cost can take into account the cost of traversing not only an edge, but a vertex, too. In routing with DPP, the cost of a path pair is minimized, and the spectrum and modulation constraints must be met for both paths.

Whether routing along paths of lowest cost leads to optimal network performance (as measured, for instance, with the bandwidth blocking probability over a series of established and terminated connections) is, to the best of our knowledge, an open research problem, which we do not address in this work. We research the problem of an exact algorithm, one which finds an optimal (i.e., of lowest cost) solution, and not of an optimal algorithm. An algorithm optimality could imply optimal network performance, or optimal computational complexity, and we address neither of these.

Our novel contribution is an algorithm which solves the dynamic routing problems *with DPP* for WDM networks and EONs without signal regeneration and spectrum conversion. The algorithm can take into account various spectrum allocation policies. With extensive simulations, we demonstrate the computational performance of the proposed algorithm, which can be polynomial, not exponential. We corroborate the optimality of the results found for networks up to 15 nodes. Finally, we provide under a liberal license our free and open-source implementation of the proposed algorithm [5].

The article is organized as follows. In Section 2, we review related works, in Section 3, we state the research problem, in Section 4, we describe the algorithm, and, in Section 5, we report on the simulation results. Finally, Section 6 concludes the article.

## 2. Related Works

The proposed algorithm is based on the *generic* Dijkstra algorithm recently proposed [6]. The generic Dijkstra algorithm is a generalization of the Dijkstra algorithm, which takes into account the spectrum continuity and contiguity constraints by introducing the *incomparability relation* between solutions. Specifically, we modify the generic Dijkstra algorithm to work on a search graph, which is built using the input graph, and represents the possible ways of finding path pairs. Furthermore, we introduce the incomparability relation between pairs of paths.

To the best of our knowledge, no efficient and exact algorithm (at least demonstrated by simulations) for solving the dynamic routing problem with DPP in optical networks has been published. In [7], the authors offered a proof that the dynamic RWA with DPP is nondeterministic polynomial time complete (NP-complete). In Reference [8], the authors offered a proof that the dynamic RMSA with DPP is NP-complete. In contrast, we propose an algorithm with the efficiency and exactness demonstrated by simulations, which suggests the problem may be tractable.

Dynamic routing without DPP is simpler, but its status seemed unclear. In References [9,10], the problem was solved with exponential worst-case time and memory complexities. However, the dynamic routing problems in EONs can be solved exactly in polynomial time with the spectrum scan method [11], introduced in Reference [12]. The exact-routing concept of the spectrum scan method was introduced earlier for WDM networks in Reference [13] but was called a *heuristic* greedy algorithm. That concept was used under the name of the spectrum window planes [14], and the filtered-graphs algorithm [6]. In Reference [15], the authors solved efficiently (in polynomial time) and exactly the dynamic routing problem in WDM networks with their interconnected-layered-graph algorithm. That *exact* algorithm was later improved and applied to EONs in Reference [16] but was called *heuristic*.

In a very broad sense, dynamic routing problems are multicriteria shortest path problems, which, in turn, are multiobjective combinatorial optimization problems with a set of constraints given to define the combinatorial structure of the problem [17]. Whether a specific routing problem is tractable or not depends on the number and type of criteria (or objective functions) and constraints. Routing problems are defined for discrete or continuous criteria: discrete for, e.g., optical networks [9] and networks capable of advance resource reservation [18], continuous for, e.g., networks with quality-of-service requirements [19,20]. In Reference [21], ten bicriteria shortest path problems were studied, some of them were proven NP-complete, others were solved in polynomial time with a novel multilabeling algorithm. That bicriteria multilabeling algorithm was generalized to any number and type of criteria in Reference [22], which is now called the *Martins algorithm*.

The Martins algorithm is the basic algorithm for exactly solving any multicriteria shortest path problem, but with exponential worst-case memory and time complexities [23]. To use the Martins algorithm for dynamic routing in optical networks, we could consider available spectrum units as discrete criteria, but that would lead to exponential worst-case time and memory complexities. The generic Dijkstra is similar to the Martins algorithm in that it is also a multilabeling algorithm, but the generic Dijkstra algorithm is a single criterion shortest path algorithm, where the ordering between solutions (labels) is partial.

The efficient and exact algorithms for finding a shortest pair of edge-disjoint paths in a graph are: the Suurballe algorithm [24], the Bhandari algorithm [25], and any minimum-cost, maximum-flow algorithm (e.g., the successive shortest path algorithm) with edge capacities set to one [26], all of which use the path augmentation technique. These algorithms cannot be used for solving the stated problem because they do not consider the spectrum continuity and contiguity constraints.

In our simulations, we also used two well-known algorithms for solving the problem: the heuristic *edge-exclusion* algorithm, and the exact *brute-force* algorithm.

The edge-exclusion algorithm is a simple and commonly-used algorithm for finding a pair of edge-disjoint paths: find a shortest path, then remove from the graph the edges found, and then find a shortest path again. This heuristic performs quite well, but it often finds suboptimal solutions, and it can fail even when a solution exists (e.g., for the so-called trap topology).

The edge-exclusion algorithm usually employs the limited (i.e., with a limited *K*, e.g., K=10) K-shortest path (KSP) algorithm to find a shortest path, which is a heuristic algorithm whose blocking probability depends on the value of *K*. However, the edge-exclusion algorithm can perform better if an algorithm of lower blocking probabilities is used. The blocking probabilities of the generic Dijkstra algorithm can be even twice as low as the blocking probabilities of the limited KSP algorithm [27]. Therefore, in the edge-exclusion algorithm, we used the generic Dijkstra algorithm.

The brute-force algorithm enumerates the path pairs using a priority queue that sorts the pairs in increasing-cost order. After we retrieve a pair from the queue, we produce new path pairs by extending one of the paths in the pair with an available edge that was not used before because the two paths should be edge-disjoint and without loops. We put a new path pair into the queue, if its paths meet the spectrum continuity and contiguity constraints. We keep looking for path pairs until we find one whose paths end at the destination node, provided we have enough time and memory. We successfully used the brute-force algorithm only for very small networks (15 nodes), since this algorithm is very inefficient.

## 3. Problem Statement

Given:

- directed multigraph G=(V,E), where $V=\{v_{i}\}$ is a set of vertexes, and $E=\{e_{i}\}$ is a set of edges,
- available units function $\mathrm{AU}(e_{i})$, which gives the set of available units of edge $e_{i}$, which do not have to be contiguous,
- *s* and *t* are the source and target vertexes of the demand,
- a cost function cost(p), which returns the cost of path *p*,
- a monotonically nondecreasing cost function COST(l), which returns the (real or integer) cost of path pair *l*,
- a decision function decide(p) of monotonically increasing requirements, which returns true if path *p* can support the demand, otherwise false,
- the set of all units Ω on every edge.

Find:

- a cheapest (i.e., of the lowest cost) pair of edge-disjoint paths (a path is a sequence of edges), the cheaper being the working path, and the more expensive the protecting path,
- continuous and contiguous units for each of the two paths separately: the working path and the protecting path (i.e., each path can have different spectrum).

We denote a set of contiguous units (CU) which start at index *a* and end at index *b* inclusive as [a..b]. For instance, [0..2] denotes units 0, 1, and 2. We can treat a set of units as a set of CUs. For instance, {0,1,3,4,5} and {[0..1],[3..5]} are the same. Two CUs are *incomparable*, when one is not included in the other. For instance, [0..2] and [2..3] are incomparable, which we denote with the ‖ relation, e.g., [0..2]‖[2..3].

To state the problem generically, we intentionally introduced the cost, COST, and decide functions to consider the RWA, RSA, and RMSA problems with DPP at once. For example, for RWA, the cost(p) function for path *p* could return the length of the path, for RSA, the product of the path length and the number of units requested by the demand, and, for RMSA, the product of the path length and the number of units required by the demand for the given path.

We require that the COST(l) function for a path pair *l* be monotonically nondecreasing, i.e., for any path pair $l^{\prime }$ derived from *l* by appending an edge to one of the paths, $\mathrm{COST}\left(l\right)\leq \mathrm{COST}\left(l^{\prime }\right)$. This requirement implies the proposed algorithm cannot be used for networks with regeneration, when the path cost is defined as the product of the path length and the required number of units. Regeneration would reduce the number of required units, and the cost of the path pair would be reduced, thus violating this requirement.

We also assume that an optimal path pair has the optimal substructure, i.e., it is built of optimal path pairs, which is required by the dynamic programming principle the proposed algorithm relies on. In our simulations, the defined problem meets this assumption: the path cost is the product of the path length and the number of units required, while the cost of a path pair is the sum of the costs of the two paths.

The decide function accepts or rejects a candidate path, and lets the user define an acceptable path. We require the function to have monotonically increasing requirements, i.e., if the function rejects path *p*, then any path derived from *p* by appending an edge should also be rejected. For RWA, the function should make sure that the CU has at least one unit (wavelength), for RSA, that the CU has at least the number of units requested by the demand, and, for RMSA, that a CU has at least the number of units required for the demand for the given path length.

The bitrate of a demand is not a given of the stated problem and, if needed, should be relegated to the decision function as an implementation detail. Likewise, the cost, COST, and decide functions remain undefined in the problem statement. In Section 5, to solve the RMSA problem with DPP, we define the cost, COST, and decide functions in Section 5.1.3. The decision function defined there by (7) checks for the required number of units, which depends on the path length.

## 4. Proposed Algorithm

We run the generic Dijkstra algorithm on a *search graph*. Searching for a cheapest solution in the search graph corresponds to searching for a pair of paths of lowest cost in the input graph. The algorithm grows *the search tree* for the search graph.

### 4.1. Preliminaries

Below, we describe the search graph, the search tree, the priority queue, and the related concepts of the solution, the path trait, and the solution label.

#### 4.1.1. Search Graph

The search graph has a set of vertexes $X=\{x=\left(v_{x,1},v_{x,2}\right)\}$, where vertex indexes $v_{x,1}$, and $v_{x,2}$ of the input graph satisfy $v_{x,1}\leq v_{x,2}$. For vertex *x*, we find a set of solutions, where the *solution* is a pair of paths: one path leads to vertex $v_{x,1}$, and the other to vertex $v_{x,2}$. Which of the paths could eventually (when vertex *t* is reached by both paths) become working or protecting is unknown and unimportant at this stage.

An edge in the search graph from vertex *x* to vertex $x^{\prime }$ represents finding a solution for vertex $x^{\prime }$ based on a solution for vertex *x* by taking edge $e^{\prime }$ in the input graph from either vertex $v_{x,1}$ or $v_{x,2}$. Therefore, the edge in the search graph connects vertex *x* to some other vertex $x^{\prime }$ which has one of the vertex indexes $v_{x,1}$ or $v_{x,2}$ taken from *x*. The other vertex index of $x^{\prime }$ is the index of the target vertex of edge $e^{\prime }$. Vertex $x^{\prime }$ becomes either $\left(v_{x^{\prime },1},v_{x,2}\right)$, or $\left(v_{x,1},v_{x^{\prime },2}\right)$, with its vertex indexes swapped if necessary, because we require the first one be smaller than or equal to the second one.

Taking a single edge in the input graph is the simplest, and the only one needed, way of producing a new solution in the search graph. Taking at once two edges of the input graph, one edge for each of the two paths, should also work, but this would lead to a more complicated and less efficient algorithm. This is more complicated because we cannot always take two edges, and less efficient because, by taking two edges, we can reach a suboptimal solution, which we would avoid if we took one of those edges first.

#### 4.1.2. Path Trait

A *path trait* *p* is a pair of a cost and a CU, which describes a path in the input graph. For a path trait *p*, function cost(p) gives the path cost, and function CU(p) the path CU. For example, assuming the cost is the path length, a path trait p=(500km,[0..10]) says the path is cost(p)=500 km long and has the CU of CU(p)=[0..10].

Path trait $p_{i}$ is better than or equal to path trait $p_{j}$, denoted by $p_{i}\leq p_{j}$, when the cost of $p_{i}$ is smaller than or equal to the cost of $p_{j}$, and the CU of $p_{i}$ includes the CU of $p_{j}$, i.e., $\left(\mathrm{cost}\left(p_{i}\right)\leq \mathrm{cost}\left(p_{j}\right)\right)\wedge \left(\mathrm{CU}\left(p_{i}\right)⊇\mathrm{CU}\left(p_{j}\right)\right)$. If $p_{i}\leq p_{j}$, then we drop $p_{j}$, since it offers no better path in comparison with $p_{i}$, and so we perform the search more efficiently.

This definition of the path trait comparison allows for *incomparability* of path traits, which is needed when searching for paths with the spectrum continuity and contiguity constraints. For instance, path trait $p_{1}=\left(1,\left[0..2\right]\right)$ is incomparable with $p_{2}=\left(2,\left[0..3\right]\right)$ because neither $p_{1}\leq p_{2}$ nor $p_{2}\leq p_{1}$ is true. We are interested in path trait $p_{2}$, even though its cost is higher than the cost of $p_{1}$, because $p_{2}$ has a CU that is incomparable with the CU of $p_{1}$.

#### 4.1.3. Solution Label

Solution label $l_{x}=\left(p_{x,1},p_{x,2}\right)$ for vertex *x* is a pair of path traits $p_{x,1}$, $p_{x,2}$, where the first path which ends at $v_{x,1}$ has trait $p_{x,1}$, and the other path which ends at $v_{x,2}$ has trait $p_{x,2}$.

We compare solution labels to drop those solutions which offer nothing better than we already have, thus limiting the search space, and performing the search more efficiently. Label $l_{i}$ is better than or equal to label $l_{j}$, denoted by $l_{i}\leq l_{j}$, when both path traits of $l_{i}$ are better than or equal to both path traits of $l_{j}$, i.e., $\left(p_{i,1}\leq p_{j,1}\right)\wedge \left(p_{i,2}\leq p_{j,2}\right)$. If $l_{i}\leq l_{j}$, then we should not be interested in $l_{j}$ because it offers no better solution in comparison with $l_{i}$.

A solution label for vertex *x* with the same vertexes in the input graph, i.e., $v_{x,1}==v_{x,2}$, should have its path traits ordered with the ≤relation, i.e., $p_{x,1}\leq p_{x,2}$, so that, when we compare labels of two solutions for vertex *x*, we compare the working-path traits first, and the equal to or worse protecting-path traits next.

This definition of the label comparison allows for *incomparability* of labels, which is needed when searching for a pair of paths, when these paths can have incomparable traits. For instance, label $l_{1}$ of path traits $p_{1,1}=\left(1,\left[0..2\right]\right)$, and $p_{1,2}=\left(2,\left[10..12\right]\right)$ is incomparable with label $l_{2}$ of path traits $p_{2,1}=\left(2,\left[0..3\right]\right)$, and $p_{2,2}=\left(10,\left[10..12\right]\right)$ because neither $l_{1}\leq l_{2}$ nor $l_{2}\leq l_{1}$ is true.

#### 4.1.4. Search Tree

The result of the search is the *search tree*, which is organized according to the dynamic-programming principle of reusing data from previous computation. Search-tree node $n_{x^{\prime }}=\left(x^{\prime },l_{x^{\prime }},e^{\prime },n_{x}\right)$ represents a solution found for the search-graph vertex $x^{\prime }=\left(v_{x^{\prime },1},v_{x^{\prime },2}\right)$ based on the solution found for node *x*. The solution is described by label $l_{x^{\prime }}=\left(p_{x^{\prime },1},p_{x^{\prime },2}\right)$: the first path of the solution which ends at $v_{x^{\prime },1}$ has trait $p_{x^{\prime },1}$, and the other path which ends at $v_{x^{\prime },2}$ has trait $p_{x^{\prime },2}$. We get the solution from the previous search-tree node $n_{x}$ for vertex *x* by taking edge $e^{\prime }$ in the input graph. For a search-tree node *n*, function label(n) returns its label, and function vertex(n) returns its search-graph vertex.

A tree node represents a solution which is either *permanent* or *tentative*. A permanent-solution node stays in the tree for good, while a tentative-solution node can be discarded. A tentative-solution node is always a leaf. A tentative solution wants to become permanent, but instead it can be discarded or never processed.

To ensure that a solution is edge-disjoint, we do not add to the search tree a solution node if its edge was already used by its ancestor in the search tree.

#### 4.1.5. Priority Queue

The optimality of the solutions found is achieved with the priority queue, which provides the cheapest solutions. The priority queue stores pairs, where a pair has a cost and a reference to a search-tree node $n_{x}$ of a tentative solution. The cost in the pair is the cost of the solution, i.e., $\mathrm{COST}(\mathrm{label}\left(n_{x}\right))$. The queue sorts the solutions in the increasing-cost order, with the cheapest solution at the top.

A tentative solution is waiting in the queue to be processed, but it also can be either discarded, if we find a better solution, or never processed, if the search finishes sooner. A tentative solution becomes permanent when it is retrieved from the queue.

### 4.2. Algorithm

The proposed algorithm has the main loop listed in Algorithm 1, and the relax procedure listed in Algorithm 2. The main loop iterates over the permanent solutions retrieved from the priority queue, while the relax procedure pushes tentative solutions to the priority queue.

The solutions for vertex *x* are maintained in the set $P_{x}$ of permanent solutions with incomparable labels, and the set $T_{x}$ of tentative solutions with incomparable labels. The set of all permanent solutions is *P*, and the set of all tentative solutions is *T*. Permanent solutions are optimal.

We start the search at vertex $x_{s}=\left(s,s\right)$. We create the tentative solution $n_{x_{s}}$ (the root of the search tree) of two empty paths starting at vertex *s* with 0 costs and the CUs of Ω. We insert $n_{x_{s}}$ into the set of tentative labels for vertex *x*, and push the pair of $\left(0,n_{x_{s}}\right)$ to the priority queue *Q*.

To cover the maximal part of the search space, we look for the paths with the maximal CU, which satisfy the requirements of the decision function decide used by the relax procedure. For this reason, we start the search with the CU of Ω.

We stop searching when the priority queue is empty, or when we find a permanent solution for vertex $x_{t}=\left(t,t\right)$. If we need a complete (i.e., for all vertexes *x*) search tree, we should let the algorithm run until the priority queue is empty.

In each iteration of the main loop, we process the cheapest of all tentative solutions, and make it permanent. When we retrieve a pair from the queue, we have to ensure the tentative solution was not discarded by the relax procedure, i.e., that the reference to $n_{x}$ is not null.

Next, we relax the out edges of vertex *x* in the search graph. An edge in the search graph represents taking an edge in the input graph from either vertex $v_{x,1}$ or $v_{x,2}$, and so we iterate over the edges leaving vertex $v_{x,1}$ first, and over the edges leaving vertex $v_{x,2}$ next.
**Algorithm 1** Dedicated Path Protection AlgorithmIn: graph *G*, source vertex *s*, target vertex *t*Out: a cheapest pair of paths, and their CUs*Here, we concentrate on permanent solutions $n_{x}$.*$x_{s}=\left(s,s\right)$$x_{t}=\left(t,t\right)$$l_{x_{s}}=\left(\left(0,\Omega \right),\left(0,\Omega \right)\right)$$n_{x_{s}}=\left(x_{s},l_{x_{s}},e_{\emptyset },\mathrm{null}\right)$$T_{x_{s}}=\left\{n_{x_{s}}\right\}$$\mathrm{push}(Q,\left(0,n_{x_{s}}\right))$**while***Q* is not empty **do**$\;\;\;\;n_{x}=\mathrm{pop}\left(Q\right)$     **if** $n_{x}==\mathrm{null}$ **then**          continue the main loop$\;\;\;\;x=\left(v_{x,1},v_{x,2}\right)=\mathrm{vertex}\left(n_{x}\right)$     // Remove $n_{x}$ from the set of tentative solutions for *x*.$\;\;\;\;T_{x}=T_{x}-\left\{n_{x}\right\}$     // Add $n_{x}$ to the set of permanent solutions for *x*.$\;\;\;\;P_{x}=P_{x}\cup \left\{n_{x}\right\}$     **if** $x==x_{t}$ **then**          break the main loop$\;\;\;\;l_{x}=\left(p_{x,1},p_{x,2}\right)=\mathrm{label}\left(n_{x}\right)$     **for each** out edge $e^{\prime }$ of vertex $v_{x,1}$ in *G* **do**$\;\;\;\;\;\;\;\;\;\mathrm{relax}(e^{\prime },v_{x,2},p_{x,2},p_{x,1},n_{x})$     **for each** out edge $e^{\prime }$ of vertex $v_{x,2}$ in *G* **do**$\;\;\;\;\;\;\;\;\;\mathrm{relax}(e^{\prime },v_{x,1},p_{x,1},p_{x,2},n_{x})$**return**$\mathrm{trace}(P,x_{t},x_{s})$

The relax procedure relaxes a single edge in the search graph, which is described in the procedure parameters: the taken edge $e^{\prime }$ in the input graph, vertex $v_{1}$ and the corresponding path trait $p_{1}$ which both do not change, the other trait $p_{2}$ of the path to which we try to append edge $e^{\prime }$, and the previous search-tree node $n_{x}$.

The relaxation can find a number of tentative solutions, which would differ only by the CU of $C^{\prime }$, because there may be a number of spectrum fragments available $\mathrm{AU}(e^{\prime })$ on edge $e^{\prime }$ which we can use for a tentative solution.

We build (if necessary, we swap the elements of pairs $x^{\prime }$ and $l_{x^{\prime }}$ with the swap function) and add a tentative solution $n_{x^{\prime }}$ to $T_{x^{\prime }}$ and *Q*, only when there is no solution with a better or equal label already found. Adding $n_{x^{\prime }}$ can make some tentative solutions invalid (since $n_{x^{\prime }}$ is better), so we discard them.
**Algorithm 2** relaxIn: edge $e^{\prime }$, const vertex $v_{1}$, const trait $p_{1}$, other trait $p_{2}$,   previous search-tree node $n_{x}$*Here, we concentrate on tentative solutions $n_{x^{\prime }}$.*$v^{\prime }=\mathrm{target}\left(e^{\prime }\right)$$c^{\prime }=\mathrm{cost}\left(\mathrm{path}\;\mathrm{of}\;p_{2}\;\mathrm{with}\;e^{\prime }\;\mathrm{appended}\right)$**for each** CU $C^{\prime }$ in $\mathrm{CU}\left(p_{2}\right)\cap \mathrm{AU}\left(e^{\prime }\right)$ **do**$\;\;\;\;x^{\prime }=\left(v_{1},v^{\prime }\right)$$\;\;\;\;p^{\prime }=\left(c^{\prime },C^{\prime }\right)$     **if** $\mathrm{decide}(p^{\prime })$ **then**$\;\;\;\;\;\;\;\;l_{x^{\prime }}=\left(p_{1},p^{\prime }\right)$         **if** $v^{\prime }<v_{1}$ **then**$\;\;\;\;\;\;\;\;\;\;\;\mathrm{swap}(x^{\prime })$$\;\;\;\;\;\;\;\;\;\;\;\mathrm{swap}(l_{x^{\prime }})$         **else if** $v_{1}==v^{\prime }$ and not $p_{1}\leq p^{\prime }$ **then**$\;\;\;\;\;\;\;\;\;\;\;\mathrm{swap}(l_{x^{\prime }})$          // Make sure we should be interested in $l_{x^{\prime }}$.          **if** $∄n\in P_{x^{\prime }}:\mathrm{label}\left(n\right)\leq l_{x^{\prime }}$ **then**               **if** $∄n\in T_{x^{\prime }}:\mathrm{label}\left(n\right)\leq l_{x^{\prime }}$ **then**                    // Make sure we are not reusing $e^{\prime }$.                    **if** $e^{\prime }$ not used by ancestors **then**                        // Discard worse tentative solutions.$\;\;\;\;\;\;\;\;\;\;\;\;\;\;\;\;\;\;\;\;\;T_{x^{\prime }}=T_{x^{\prime }}-\left\{n\in T_{x^{\prime }}:l_{x^{\prime }}\leq \mathrm{label}\left(n\right)\right\}$$\;\;\;\;\;\;\;\;\;\;\;\;\;\;\;\;\;\;\;\;\;n_{x^{\prime }}=\left(x^{\prime },l_{x^{\prime }},e^{\prime },n_{x}\right)$                        // Add $n_{x^{\prime }}$ to the tentative solutions for $x^{\prime }$.$\;\;\;\;\;\;\;\;\;\;\;\;\;\;\;\;\;\;\;\;\;T_{x^{\prime }}=T_{x^{\prime }}\cup \left\{n_{x^{\prime }}\right\}$$\;\;\;\;\;\;\;\;\;\;\;\;\;\;\;\;\;\;\;\;\;\mathrm{push}(Q,\left(\mathrm{COST}\left(l_{x^{\prime }}\right),n_{x^{\prime }}\right))$

Spectrum allocation policy should be taken into account in two places. First, the priority queue should choose the solution of the preferred spectrum allocation policy from among the tentative solutions of the same cost. Second, once the solution for the destination vertex is found, the preferred CUs should be allocated from the CUs found.

The trace function, using *P*, traces back the tree nodes from the node for vertex $x_{t}$ to the node for vertex $x_{s}$. For each tree node there is an edge, which the function appends to one of the two paths. When appending an edge to a path, we have to ensure that not only the cost matches, but the CU matches, too. We have to consult the other path trait of the tree node, to ensure that we are not appending the edge to the wrong path, which coincidentally meets the conditions. The function returns the less expensive path as the working path, and the more expensive path as the protecting. For each of the paths, the function allocates the minimal CU, with the required number of units, from the maximal CU found for the permanent solution for vertex $x_{t}$.

### 4.3. Example

**Figure 1:**
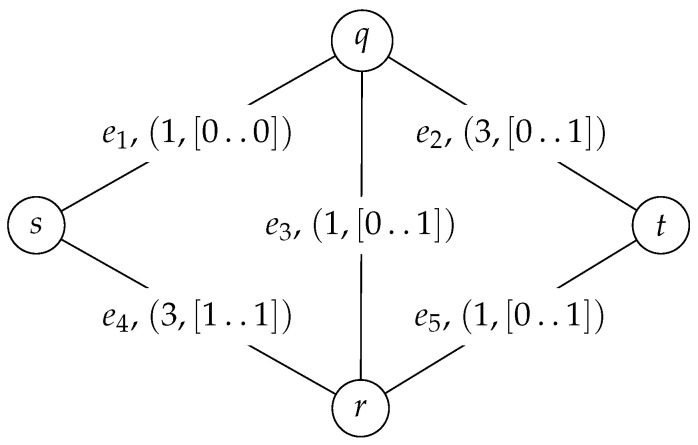
A sample input graph: the trap topology.

We demonstrate how the algorithm works by finding a solution from the source vertex *s* to the destination vertex *t* for a single unit in the trap topology shown in Figure 1, where an edge label gives not only the name of the edge, but also its cost and available units. Not to complicate the example further, the signal modulation constraints are not considered, especially since they are not crucial to the algorithm as they only discard paths.

The trap topology is well-known since the edge-exclusion algorithm fails for it: when the edges of the shortest path from *s* to *t* through vertexes *q* and *r* are removed, a second path does not exist. However, the *optimal solution* does exist: one path goes through vertex *q* with the CU of [0..0], and the other through vertex *r* with [1..1].

**Figure 2:**
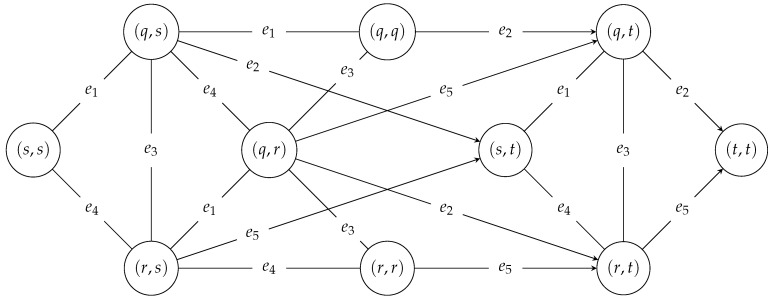
The search graph.

Figure 2 shows the search graph generated, where the edge label gives the name of an input-graph edge to take to make a transition between the vertexes in the search graph. For example, the transition from vertex (s,s) to vertex (q,s) requires edge $e_{1}$. Most edges are undirected, since the algorithm examines transitions in both directions. However, there are some directed edges (e.g., from (q,s) to (s,t)), since their reverse transitions (e.g., from (s,t) to (q,s)) are not examined. Because paths in the input graph have to be edge-disjoint, some paths in the search graph are disallowed, e.g., (s,s)−(q,s)−(q,q).

**Figure 3:**
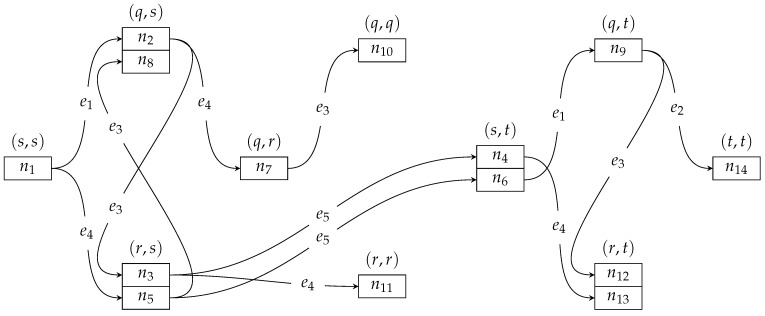
The search tree.

Figure 3 shows the search tree generated, where only the permanent (and not tentative) solutions are represented. The tree is rooted at node $n_{1}$ for vertex (s,s). The solution found is represented by node $n_{14}$ for vertex (t,t). A search-graph vertex can have a set of permanent incomparable solutions, which are represented by a set of search-tree nodes, e.g., for vertex (q,s) there are two search-tree nodes: $n_{2}$ and $n_{8}$.

The algorithm processes solution labels by taking actions on them, as reported in Table 1. A label can be produced and pushed into the queue, as in, e.g., action #0. A row represents an action on a label which was produced for the given search-graph vertex by making a transition with the given edge. For instance, action #2 reports a label that is pushed into the queue, and which was produced for the search-graph vertex (q,s) by making a transition with edge $e_{1}$ (from vertex (s,s)).

A label can be retrieved from the queue, and made permanent, as in, e.g., action #1. A search-tree node for a permanent label has a name reported, e.g., $n_{1}$ as in action #1. A row for a label made permanent is colored gray to mark the beginning of a sequence of rows that report the actions of the relaxation based on the label from that gray row. For instance, the row for action #1 is gray, and the subsequent rows for actions from #2 to #5 report the actions of the relaxation that ensued.

A label can be pushed into the queue, or it can be dropped for two reasons: it is worse than or equal to an existing label (e.g., in action #4, a label is dropped because an equal label of action #2 exists), or it uses an edge twice (e.g., in action #7, a label is dropped because edge $e_{1}$ is used twice). A label can also be discarded from the queue if a better label is found (e.g., in action #17, a label is discarded because a better label was found in action #16).

As reported in Table 1, the search is booted with action #0. There are 14 permanent labels found for 10 vertexes of the search graph. The algorithm terminates, when the destination node (t,t) is reached with the search-tree node $n_{14}$. We can trace back from $n_{14}$ to $n_{1}$ to get the aforementioned optimal solution.

### 4.4. Worst-Case Analysis

We argue the size of the search space is polynomially upper bounded. We derive the upper bound *L* of the number of incomparable labels (i.e., the size of the search space) by considering the worst case where every vertex of all |X| search graph vertexes has the maximum number *S* of incomparable labels. Therefore, L=|X|S. The problem is to derive |X| and *S*.

The number of vertexes in the search graph is given by (1), since the input graph has |V| vertexes, and since vertexes $x=(v_{x,1},v_{x,2})$ of the search graph satisfy $v_{x,1}\leq v_{x,2}$. The number of vertexes with $v_{x,1}=v_{x,2}$ is |V|, and the number of vertexes with $v_{x,1}<v_{x,2}$ is the number of combinations of two elements from the set of |V| elements.

(1) |X|=|V|+|V|2=|V|(|V|+1)2.

The maximum number *S* of incomparable labels a vertex can have is given by (2). Since a label describes a solution made up of two independent paths, *S* is the maximum number of incomparable path traits squared.

The maximum number of incomparable path traits depends only on Ω. We get the largest set of incomparable path traits when the cost of path traits increases as the size of their CUs increases. The largest set has |Ω| subsets: the first subset has |Ω| traits with CUs of a single unit and the lowest cost, the second subset has |Ω|−1 traits with CUs of two units and a higher cost, …, and the last subset has a single trait with the CU of |Ω| units and the highest cost. The largest set has 1+2+…+|Ω|=(|Ω|+1)|Ω|/2 incomparable path traits.

(2) $$S={\left(\frac{\left(|\Omega |+1)|\Omega \right|}{2}\right)}^{2}.$$

Therefore, the size of the search space is polynomially upper bounded, since $O\left(L\right)=O(|V{|}^{2}|\Omega {|}^{4})$.

## 5. Simulations

The simulations had two goals: the optimality corroboration, and the performance evaluation. We had 48,600 simulation runs: 32,400 corroborative runs, and 16,200 performance evaluation runs.

We corroborated the optimality of the results of our algorithm by comparing them with the results of the brute-force enumeration algorithm: for a single search either both algorithms returned results of the same cost, or both algorithms returned no results. Since there are billions of feasible solutions even in small networks, and since the brute-force algorithm enumerates them all, we were able to corroborate the results only for small networks.

We ran the corroborative simulations for networks of 10, 11, 12, 13, 14, and 15 nodes; for 160, 320, and 640 units; for offered loads ranging from light to heavy; and for demands requesting on average from 10 to 64 units. In total, we had 32,400 simulation runs, out of which 183 runs were killed, because they requested more than 120 GB of operating memory, which we did not have. In total, we carried out 17,590,624 searches, all successfully corroborated.

The remainder of this section is about the performance evaluation.

### 5.1. Simulation Setting

Below, we describe how we model the network, the traffic, and the signal modulation.

#### 5.1.1. Network Model

A network model has an undirected graph, and |Ω|. We randomly generated three groups of network graphs with 25, 50, and 100 vertexes, where each group had one hundred graphs. We generated Gabriel graphs because they have been shown to model the properties of the transport networks very well [28]. The vertexes were uniformly distributed over a square area with the density of one vertex per 10 thousand square km.

We used three spacings of 25 GHz, 12.5 GHz, and 6.25 GHz for the erbium band, which translated to three values for |Ω|: 160, 320, and 640 units.

We present the results only for the first-fit spectrum allocation policy. The first-fit policy allocates units in the first CU that can support the demand, i.e., the CU with the units of the lowest indexes. We also considered the best-fit and random-fit policies. The best-fit policy performed comparably to the first-fit policy, and the random-fit policy performed markedly worse than the first-fit policy. We do not present the results for these alternative policies because they add little to the main results.

#### 5.1.2. Traffic Model

We evaluate the algorithm performance as a function of the *network utilization*, which we define as the ratio of the number of units in use to the total number of units on all edges. We measure the network utilization in response to offered load *a*, which expresses the desired network utilization.

Demands arrive according to the exponential distribution with rate λ per day. The end nodes of a demand are different and chosen at random. The number of units a demand requests is described by distribution (Poisson(γ−1)+1) with the mean of γ, i.e., a shifted Poisson distribution, so that we do not get a zero. Parameter $\gamma_{p}$ expresses the mean number of units that demands request relative to the number |Ω| of all units on every edge, and so $\gamma =\gamma_{p}\left|\Omega \right|$. We model the connection holding time with the exponential distribution with the mean of τ days. A connection is bidirectional: the same CU is allocated in both directions for a path.

We express λ as a function of *a*. The offered load is the ratio of the number of demanded units to the total number of units on all edges. For traffic intensity λτ, the number of units demanded is 2λτγα, since a demand requests two paths, and we estimate they require γ units, and α edges each, where α is the average number of edges of a shortest path between the end nodes of the demand in the network being simulated. Therefore, a=2λτγα/|E||Ω|, from which (3) follows.

(3) $$\lambda \left(a\right)=\frac{a|E||\Omega |}{2\tau \gamma \alpha }.$$

Equation (3) underestimates the value of λ(a) because we assume that every demand has a connection established. For this reason, a=1 does not yield a full network utilization.

#### 5.1.3. Signal Modulation Model

We use the signal modulation model from Reference [6], with *M* modulations available. For a demand requesting *g* units for the most spectrally-efficient modulation, the number of units needed to establish a connection of length *d* is given by (4), where $r_{1}$ is the reach of the least spectrally-efficient modulation, and $r_{M}$ is the reach of the most spectrally-efficient modulation.

(4) $$u\left(g,d\right)=\left\{\begin{array}{cc} g & \mathrm{if}\;d\leq r_{M}\\ \infty & \mathrm{if}\;r_{1}<d\\ \left\lceil g\cdot log_{2}\left(2d/r_{M}\right)\right\rceil & \mathrm{otherwise} \end{array}\right..$$

We describe a demand with the number of units *g*, instead of bitrate *b*, because the algorithm works with units, not bitrates. If the bitrate is given, we can calculate the number of units using (5), where *R* is a technology-dependent bitrate (e.g., 2.5 Gb/s), and *G* is the number of guard-band units [29].

(5) g(b)=⌈b/(R·M)⌉+G.

In the simulations, we assumed M=4, and the reach of the least-spectrally efficient modulation $r_{1}$ equals to one and a half lengths of the longest path from among all the shortest paths (i.e., for every source-destination combination) in the network being simulated, which allows us to consider paths much longer than an average shortest path. Following Reference [6], we calculated $r_{M}=r_{1}/2^{M-1}$.

#### 5.1.4. The Cost and Decision Functions

The cost and decision functions for path *p* are given by (6) and (7), where function length returns the length of path *p* as the sum of positive lengths of the edges used. The cost function for path pair *l* is given by (8).

(6) cost(p)=length(p)·u(g,length(p)),

(7) decide(p)=u(g,length(p))≤|CU(p)|,

(8) $$\mathrm{COST}\left(l=\left(p_{1},p_{2}\right)\right)=\mathrm{cost}\left(p_{1}\right)+\mathrm{cost}\left(p_{2}\right).$$

### 5.2. Runs and Populations

*A simulation run* simulated 150 days of a network in operation, with the results from the first 50 days discarded. The parameters of a simulation run were: the network size, |Ω|, γ, *a*, and τ. A simulation run reported the mean network utilization, the mean and maximum times taken, and the mean and maximum number of 64-bit memory words used by a search for a single demand.

We averaged the mean simulation results to calculate the *sample* mean results, which estimate the *population* mean results, and the average algorithm performance. We took the maximum of the maximum simulation results to get the *sample* maximum results, which estimate the population maximum results, and the worst-case algorithm performance.

In a given population, there were 100 simulation runs whose parameters differed only with the network model. We had 162 populations because we varied 3 network sizes (25, 50, 100 nodes), 3 values of |Ω| (160, 320, 640 units), 9 values of *a* (0.05, 0.1, 0.15, 0.2, 0.45, 0.65, 1, 1.5, 2), and two runs for γ=10 units, and $\gamma_{p}=10\%$ of units available (i.e., 16 units for the case with 160 units, 32 for 320, and 64 for 640). For all populations, the mean connection holding time τ=10 days was constant. In total, we carried out 16200 simulation runs (162 populations × 100 samples) with 24,043,157 searches. The sample means credibly estimate the population means, since their relative standard error was below 5%.

### 5.3. Simulation Results

**Figure 4:**
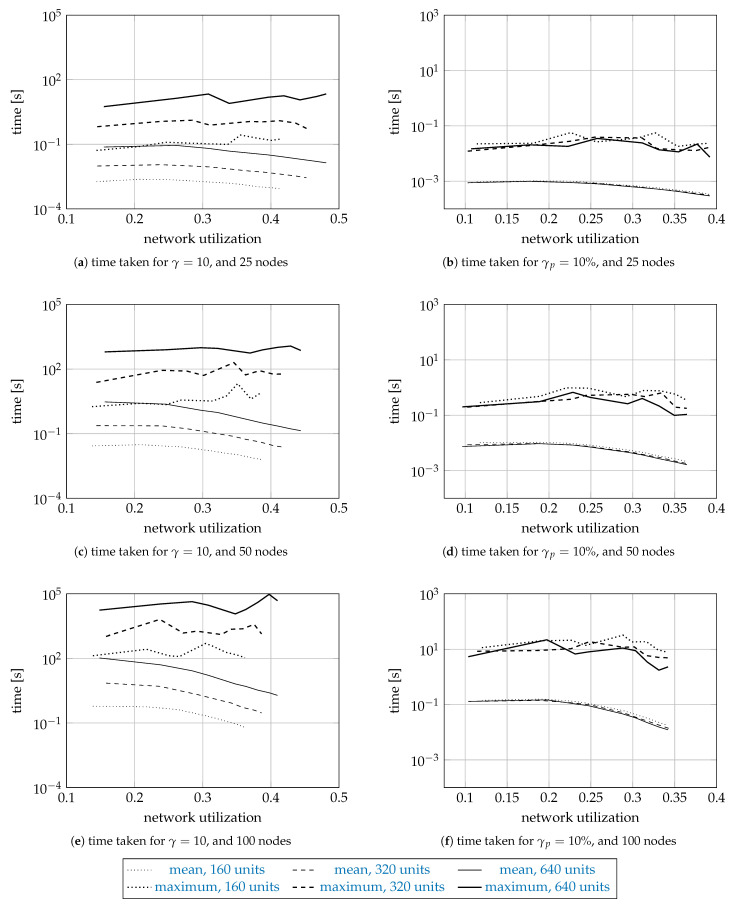
The sample means and maxima of the time taken by the proposed algorithm.

**Figure 5:**
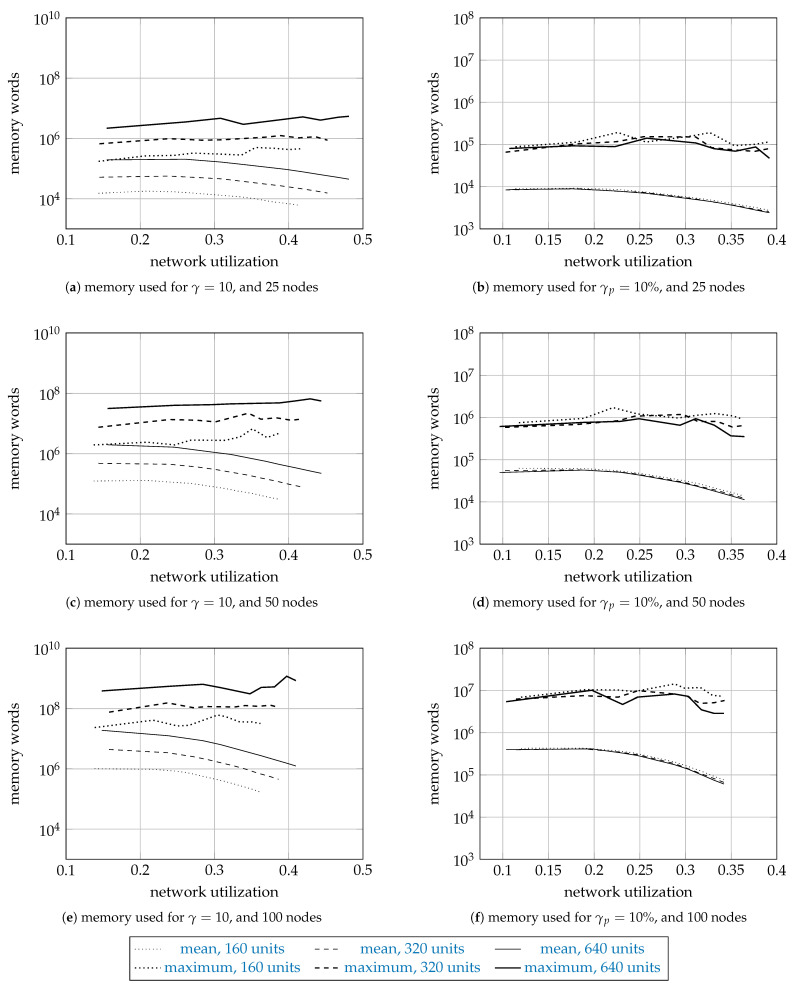
The sample means and maxima of the memory used by the proposed algorithm.

Figure 4 and Figure 5 show the sample means and the sample maxima of the time taken and memory used by a search, regardless of whether the search was successful or not. The results are shown on a logarithmic scale as a function of network utilization. The curves are plotted dotted for 160 units, dashed for 320 units, and solid for 640 units. The sample means are plotted thin, and the sample maxima thick. Each curve is drawn using 9 data points for different values of *a*. For the means, we do not report the error bars representing the standard error, since they were too small to plot.

Figure 4 and Figure 5 have three rows and two columns of subfigures. The first row shows the results for the networks with 25 nodes, the second for 50 nodes, and the third for 100 nodes. The first column shows the results for γ=10, and the second column the results for $\gamma_{p}=10\%$.

The mean times range from $10^{-3}$ s (for 25 nodes, and 160 units) to $10^{2}$ s (100 nodes, 640 units). While the difference in scale is $10^{5}$, we also note that the problem size increased 16 times. The mean time increases about ten times as we increase the network size by a factor of two. For γ=10, the mean time increases about five times as the number of units increases twice (from 160 to 320, and from 320 to 640 units). Interestingly, the time for $\gamma_{p}=10\%$ is roughly the same for 160, 320, and 640 units, which suggests the time complexity depends on the number of units requested relative to the number of available units, and indirectly on the spectrum fragmentation. The mean time decreases as the network utilization increases, since the search space gets smaller. As for the sample maximum results, they were usually a hundred times larger than the mean results.

The memory results report the number of 64-bit memory words used by the permanent solutions, the tentative solutions, and the priority queue. The network size, |Ω|, and γ affected the memory results similar to how they affected the time results. For the networks with 25 nodes, the mean number of words was about $10^{5}$, while, for the networks with 100 nodes, about $10^{9}$.

The memory used for γ=10 is far larger than for $\gamma_{p}=10\%$ because the spectrum (the available units) is more fragmented (since it is allocated in smaller fragments), and the algorithm finds more solutions as the search space is larger. Finding more solutions requires more time: simulations for γ=10 take more time that the simulations for $\gamma_{p}=10\%$.

**Figure 6:**
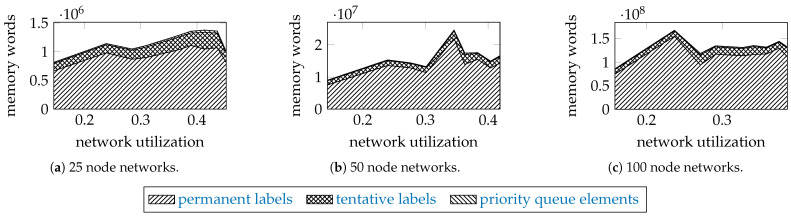
Simulation results: the maximum number of required words for networks with 320 units, and γ=10.

To examine how the incomparable permanent and tentative labels, and the elements of the priority queue contribute to the memory usage, Figure 6 shows as stack plots the maximal memory used by the proposed algorithm for the networks of 25, 50, and 100 nodes with 320 units, and γ=10.

The permanent labels take about 80% of memory, the tentative labels about 20%, and the elements of the priority queue take only a small fraction. We assumed that a label takes 15 64-bit words (implementation details: 4 words for a shared pointer, 1 word for a vertex pair, 4 words for a path trait, 2 words for an edge, 4 words for a shared pointer to the parent node in the search tree). An element of a priority queue is two words long (1 word for cost, 1 word for a weak pointer to its tentative label).

Most of the memory required by the algorithm is consumed by the permanent labels because of the large search space. A permanent label stores an optimal solution, and the results suggest that there are many of them for large networks. Furthermore, that large number of permanent labels helps to keep the number of tentative labels relatively much smaller through the edge relaxation.

**Figure 7:**
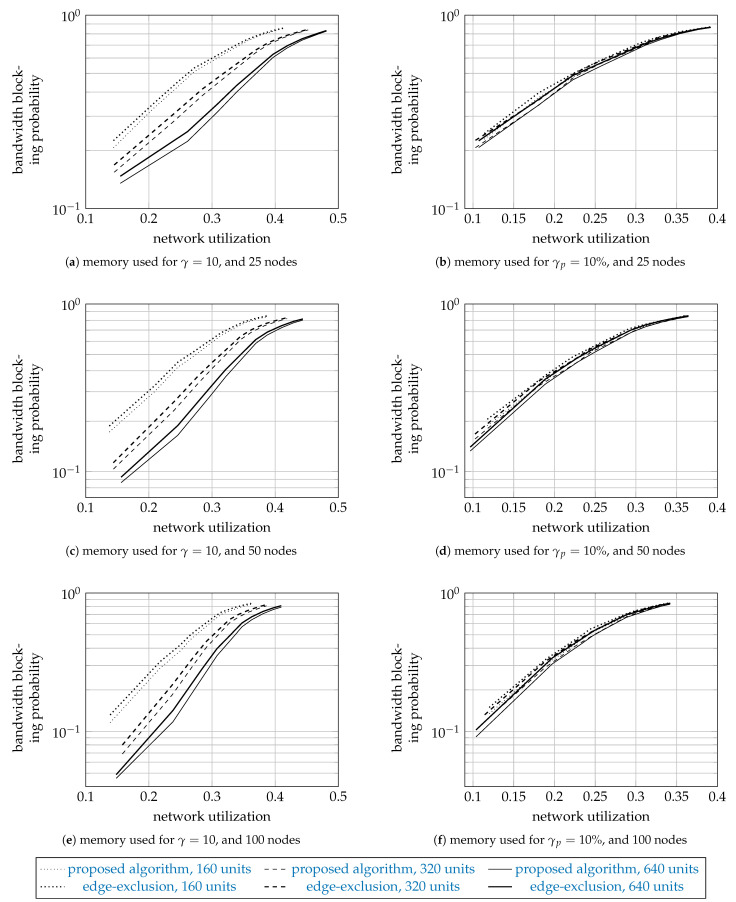
Simulation results: the sample means of the bandwidth blocking probability.

To further validate the proposed algorithm, Figure 7 shows, for all populations of interest, the mean bandwidth blocking probabilities of the proposed algorithm as thin curves and of the edge-exclusion algorithm as thick curves. The figure has two rows and three columns of subfigures. The first row shows the results for γ=10, and the second for $\gamma_{p}=10\%$. The first column shows the results for the networks with 25 nodes, the second for 50 nodes, and the third for 100 nodes. We do not plot the error bars representing the standard error, since they were too small to plot.

Since the proposed algorithm can be exact, and the edge-exclusion algorithm is heuristic, the proposed algorithm should perform better, and indeed this is so. Interestingly, the edge-exclusion algorithm (which uses the generic Dijkstra algorithm) performs very well, at only about 5% worse.

We did not add the edge-exclusion algorithm to the time and memory performance comparison, since it is a heuristic algorithm with the worst-case computational complexity of the dynamic routing problem without DPP, i.e., $O(|\Omega {|}^{2}|V|log|V|)$ [6]. In addition, we were unable to add the brute-force algorithm to the comparison because of its exponential complexity.

Admittedly, the reported time and memory consumption of the proposed algorithm seems large: for a network of 100 nodes and 640 units, the algorithm can run even for thousands of seconds, and use even 10 GBs of operating memory. However, to put these results in perspective, we report that, for a *far smaller* network of 15 nodes and 640 units, the brute-force algorithm ran for thousands of seconds, and it requested more than 120 GBs of operating memory in the corroborative runs.

## 6. Conclusions

The proposed algorithm is capable of solving various dynamic routing problems with dedicated path protection in optical networks, but not all of them, e.g., the algorithm cannot be applied when signal regeneration or spectrum conversion are used. However, the proposed algorithm can solve those routing problems that meet the minimal requirements of the stated research problem. If not, then perhaps the proposed algorithm and its novel principles could be used as a basis for devising more capable algorithms.

The proposed algorithm can also be used to find a pair of paths to different (primary, and secondary) data centers. The algorithm could even be useful in routing with inverse multiplexing, and multipath routing.

We are unable to compare the performance results of the proposed algorithm to some efficient and exact algorithm because, to the best of our knowledge, *no such competing algorithm exists*. For that large problem size, we could not have used the existing exact methods (e.g., the brute-force algorithm, integer linear programming) since they are inefficient, nor could we have used the existing efficient methods (e.g., the edge-exclusion algorithm, the tabu search), since they are suboptimal.

Dedicated path protection can be implemented at the multiplex section (fiber protection), the optical layer (optical signal protection), or the digital layer (the digital signal protection). We presented our algorithm in the setting of the optical signal protection, where we take into account the spectrum continuity and contiguity constraints, but the same principles could be used for other layers (e.g., the Internet protocol layer with multiprotocol label switching) and networks, too.

The algorithm can be adapted for further constraints, e.g., node-disjoint paths or the same spectrum fragments on both paths. Node-disjoint paths can be found if we do not relax the edges of the search graph that leave vertex $\left(v_{x,1},v_{x,2}\right)$ when $v_{x,1}==v_{x,2}$. The same spectrum fragment on both paths can be enforced by making sure during edge relaxation that the intersection of two fragments meet the requirements of a demand.

Future work could concentrate on applying the algorithm to related problems (e.g., establishing protected content-oriented connections to data centers), and further improving its performance with parallel computing.

Furthermore, perhaps the principle of the incomparability of solutions could be applied to the path augmentation technique, thus making, e.g., the Suurballe algorithm, even faster than the proposed algorithm.

The provided implementation does not require proprietary software, is implemented in modern C++ using the Boost Graph Library, and with modern functionality, such as concepts, smart pointers, in-place object creation, and move semantics. The implementation can be used to replicate the presented results, as well as stress-test the proposed algorithm.

## Figures and Tables

**Table 1:** Solution labels processed.

Action Number	Solution Cost	Search-Tree Node Name	Search-Graph Vertex	Solution Label	Edge	Action
0	0		(s,s)	((0,[0..1]),(0,[0..1]))	$e_{\emptyset }$	push into queue
1	0	$n_{1}$	(s,s)	((0,[0..1]),(0,[0..1]))	$e_{\emptyset }$	make permanent
2	1		(q,s)	((1,[0..0]),(0,[0..1]))	$e_{1}$	push into queue
3	3		(r,s)	((3,[1..1]),(0,[0..1]))	$e_{4}$	push into queue
4	1		(q,s)	((1,[0..0]),(0,[0..1]))	$e_{1}$	drop (worse or equal)
5	3		(r,s)	((3,[1..1]),(0,[0..1]))	$e_{4}$	drop (worse or equal)
6	1	$n_{2}$	(q,s)	((1,[0..0]),(0,[0..1]))	$e_{1}$	make permanent
7	2		(q,q)	((1,[0..0]),(1,[0..0]))	$e_{1}$	drop (edge reuse)
8	4		(q,r)	((1,[0..0]),(3,[1..1]))	$e_{4}$	push into queue
9	2		(s,s)	((0,[0..1]),(2,[0..0]))	$e_{1}$	drop (worse or equal)
10	4		(s,t)	((0,[0..1]),(4,[0..0]))	$e_{2}$	push into queue
11	2		(r,s)	((2,[0..0]),(0,[0..1]))	$e_{3}$	push into queue
12	2	$n_{3}$	(r,s)	((2,[0..0]),(0,[0..1]))	$e_{3}$	make permanent
13	3		(q,r)	((1,[0..0]),(2,[0..0]))	$e_{1}$	drop (edge reuse)
14	5		(r,r)	((2,[0..0]),(3,[1..1]))	$e_{4}$	push into queue
15	3		(q,s)	((3,[0..0]),(0,[0..1]))	$e_{3}$	drop (worse or equal)
16	3		(s,t)	((0,[0..1]),(3,[0..0]))	$e_{5}$	push into queue
17	4		(s,t)	((0,[0..1]),(4,[0..0]))	$e_{2}$	discard from queue
18	3	$n_{4}$	(s,t)	((0,[0..1]),(3,[0..0]))	$e_{5}$	make permanent
19	4		(q,t)	((1,[0..0]),(3,[0..0]))	$e_{1}$	drop (edge reuse)
20	6		(r,t)	((3,[1..1]),(3,[0..0]))	$e_{4}$	push into queue
21	3	$n_{5}$	(r,s)	((3,[1..1]),(0,[0..1]))	$e_{4}$	make permanent
22	4		(q,r)	((1,[0..0]),(3,[1..1]))	$e_{1}$	drop (worse or equal)
23	6		(r,r)	((3,[1..1]),(3,[1..1]))	$e_{4}$	drop (edge reuse)
24	4		(q,s)	((4,[1..1]),(0,[0..1]))	$e_{3}$	push into queue
25	6		(s,s)	((0,[0..1]),(6,[1..1]))	$e_{4}$	drop (worse or equal)
26	4		(s,t)	((0,[0..1]),(4,[1..1]))	$e_{5}$	push into queue
27	4	$n_{6}$	(s,t)	((0,[0..1]),(4,[1..1]))	$e_{5}$	make permanent
28	5		(q,t)	((1,[0..0]),(4,[1..1]))	$e_{1}$	push into queue
29	7		(r,t)	((3,[1..1]),(4,[1..1]))	$e_{4}$	drop (edge reuse)
30	4	$n_{7}$	(q,r)	((1,[0..0]),(3,[1..1]))	$e_{4}$	make permanent
31	5		(r,s)	((3,[1..1]),(2,[0..0]))	$e_{1}$	drop (worse or equal)
32	7		(r,t)	((3,[1..1]),(4,[0..0]))	$e_{2}$	drop (worse or equal)
33	5		(r,r)	((2,[0..0]),(3,[1..1]))	$e_{3}$	drop (worse or equal)
34	5		(q,q)	((1,[0..0]),(4,[1..1]))	$e_{3}$	push into queue
35	7		(q,s)	((1,[0..0]),(6,[1..1]))	$e_{4}$	drop (worse or equal)
36	5		(q,t)	((1,[0..0]),(4,[1..1]))	$e_{5}$	drop (worse or equal)
37	4	$n_{8}$	(q,s)	((4,[1..1]),(0,[0..1]))	$e_{3}$	make permanent
38	5		(q,q)	((1,[0..0]),(4,[1..1]))	$e_{1}$	drop (worse or equal)
39	7		(q,r)	((4,[1..1]),(3,[1..1]))	$e_{4}$	drop (edge reuse)
40	7		(s,t)	((0,[0..1]),(7,[1..1]))	$e_{2}$	drop (worse or equal)
41	5		(r,s)	((5,[1..1]),(0,[0..1]))	$e_{3}$	drop (worse or equal)
42	5	$n_{9}$	(q,t)	((1,[0..0]),(4,[1..1]))	$e_{1}$	make permanent
43	6		(s,t)	((2,[0..0]),(4,[1..1]))	$e_{1}$	drop (worse or equal)
44	8		(t,t)	((4,[0..0]),(4,[1..1]))	$e_{2}$	push into queue
45	6		(r,t)	((2,[0..0]),(4,[1..1]))	$e_{3}$	push into queue
46	5	$n_{10}$	(q,q)	((1,[0..0]),(4,[1..1]))	$e_{3}$	make permanent
47	6		(q,s)	((4,[1..1]),(2,[0..0]))	$e_{1}$	drop (worse or equal)
48	8		(q,t)	((4,[1..1]),(4,[0..0]))	$e_{2}$	push into queue
49	6		(q,r)	((4,[1..1]),(2,[0..0]))	$e_{3}$	drop (edge reuse)
50	8		(q,t)	((1,[0..0]),(7,[1..1]))	$e_{2}$	drop (worse or equal)
51	6		(q,r)	((1,[0..0]),(5,[1..1]))	$e_{3}$	drop (worse or equal)
52	5	$n_{11}$	(r,r)	((2,[0..0]),(3,[1..1]))	$e_{4}$	make permanent
53	6		(q,r)	((3,[0..0]),(3,[1..1]))	$e_{3}$	drop (worse or equal)
54	6		(r,t)	((3,[1..1]),(3,[0..0]))	$e_{5}$	drop (worse or equal)
55	6		(q,r)	((4,[1..1]),(2,[0..0]))	$e_{3}$	drop (edge reuse)
56	8		(r,s)	((2,[0..0]),(6,[1..1]))	$e_{4}$	drop (worse or equal)
57	6		(r,t)	((2,[0..0]),(4,[1..1]))	$e_{5}$	drop (worse or equal)
58	6	$n_{12}$	(r,t)	((2,[0..0]),(4,[1..1]))	$e_{3}$	make permanent
59	7		(q,t)	((3,[0..0]),(4,[1..1]))	$e_{3}$	drop (worse or equal)
60	7		(t,t)	((3,[0..0]),(4,[1..1]))	$e_{5}$	drop (edge reuse)
61	6	$n_{13}$	(r,t)	((3,[1..1]),(3,[0..0]))	$e_{4}$	make permanent
62	7		(q,t)	((4,[1..1]),(3,[0..0]))	$e_{3}$	drop (edge reuse)
63	9		(s,t)	((6,[1..1]),(3,[0..0]))	$e_{4}$	drop (worse or equal)
64	7		(t,t)	((3,[0..0]),(4,[1..1]))	$e_{5}$	drop (edge reuse)
65	8	$n_{14}$	(t,t)	((4,[0..0]),(4,[1..1]))	$e_{2}$	make permanent
